# Supplementary material for: Creating tunable lateral optical forces through multipolar interplay in single nanowires
Source: Nat Commun. 2023 Oct 11;14:6361. doi: 10.1038/s41467-023-42076-x (PMC10567843; doi:10.1038/s41467-023-42076-x)
Supplement: Supplementary file 1 — Supplementary Information [file 41467_2023_42076_MOESM1_ESM.pdf]

Creating tunable lateral optical forces through multipolar interplay in single nanowires

Fan Nan[1]*, Francisco J. Rodríguez-Fortuño[2], Shaohui Yan[3]*, Jack J. Kingsley-Smith[2], Jack Ng[4], Baoli Yao[3], Zijie Yan[5†] and Xiaohao Xu[3]*

[1]Guangdong Provincial Key Laboratory of Nanophotonics Manipulation, Institute of Nanophotonics, Jinan University, Guangzhou 511443, China

[2]Department of Physics and London Centre for Nanotechnology, King's College London, WC2R 2LS, United Kingdom

[3]State Key Laboratory of Transient Optics and Photonics, Xi'an Institute of Optics and Precision Mechanics, Chinese Academy of Sciences, Xi'an, 710119, China

[4]Department of Physics, Southern University of Science and Technology, Shenzhen, Guangdong 518055, China

[5]Department of Applied Physical Sciences, University of North Carolina at Chapel Hill, Chapel Hill, North Carolina 27599, United States

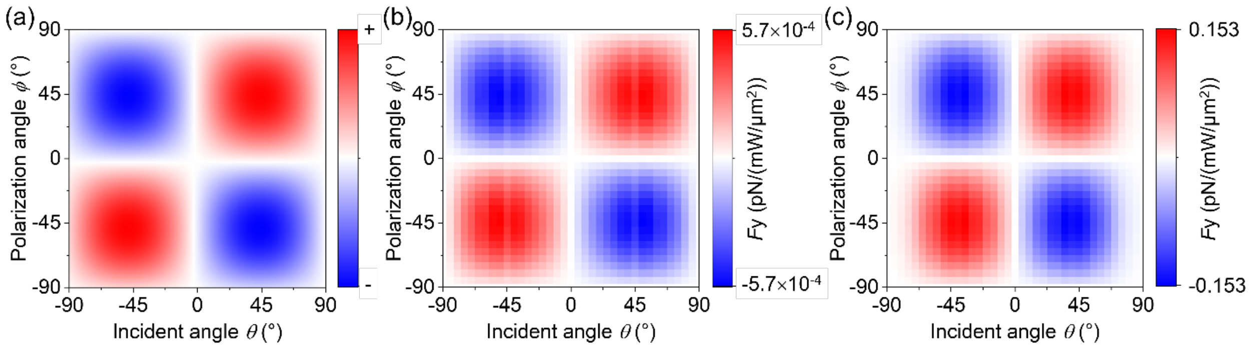

Supplementary Figure 1. The incident and polarization angle-dependent LOF. (a) The analytical results in the dipole approximation (Eq. 5). (b) Calculated LOF of a short Ag nanocylinder (L = 100 nm) using the finite-difference time-domain (FDTD) method. (c) Calculated LOF of a long Ag nanocylinder (L = 3900 nm) using the FDTD method. The diameter of the Ag nanocylinder is 80 nm.

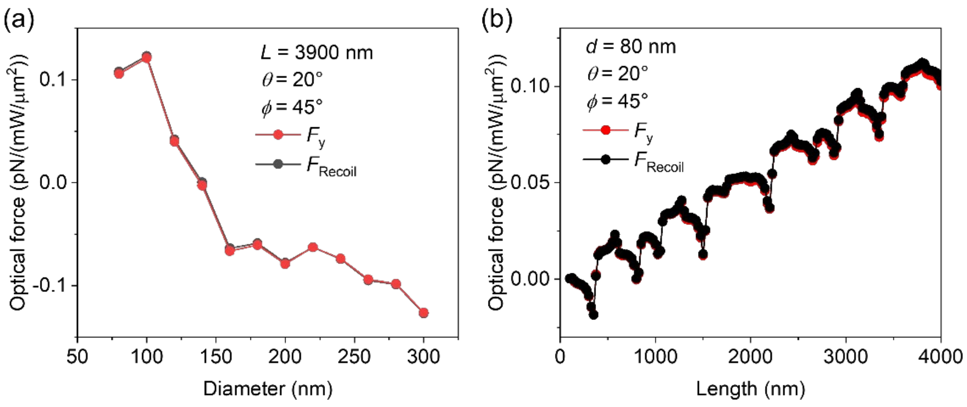

Supplementary Figure 2. LOF arising from photon recoil. Calculated recoil force (F_{Recoil}) in a series of Ag nanocylinder with (a) different diameters and (b) lengths and comparison to the corresponding LOF (F_y).

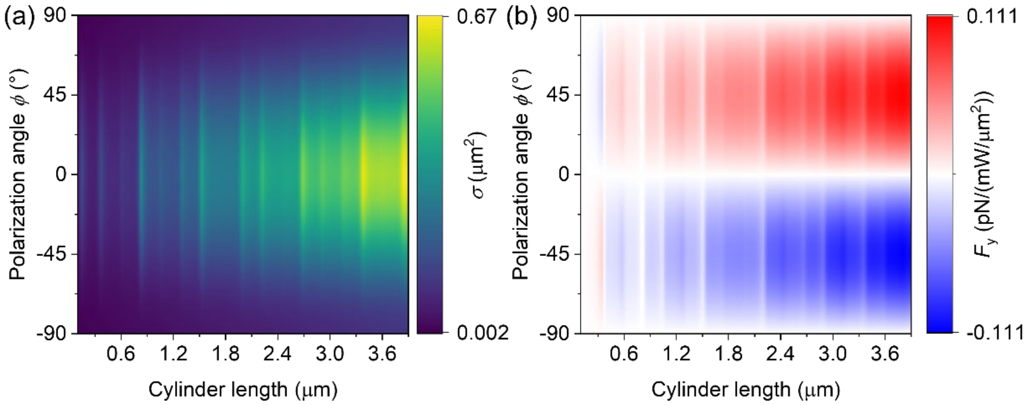

Supplementary Figure 3. LOF versus the polarization angle and cylinder length. (a) Calculated polarization angle-resolved scattering cross sections of a Ag cylinder with different lengths. The abrupt changes shown in the scattering spectrum are caused by multipolar effects. (b) Calculated LOFs of the Ag cylinder with different lengths. The diameter of the cylinder is 80 nm. The incident angle is fixed at 20°.

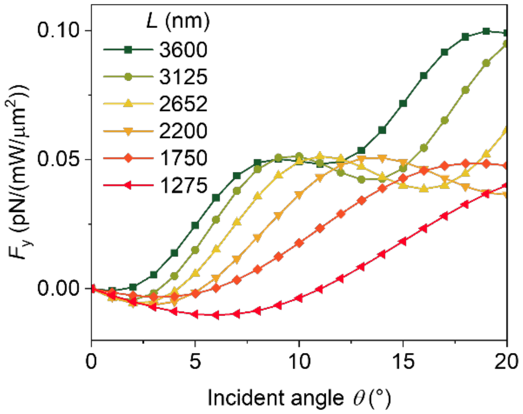

Supplementary Figure 4. Calculated incident angle dependent LOF on a series of Ag nanocylinders with different lengths. Crossover from negative to positive LOF occurs at certain combinations of the length and incident angle.

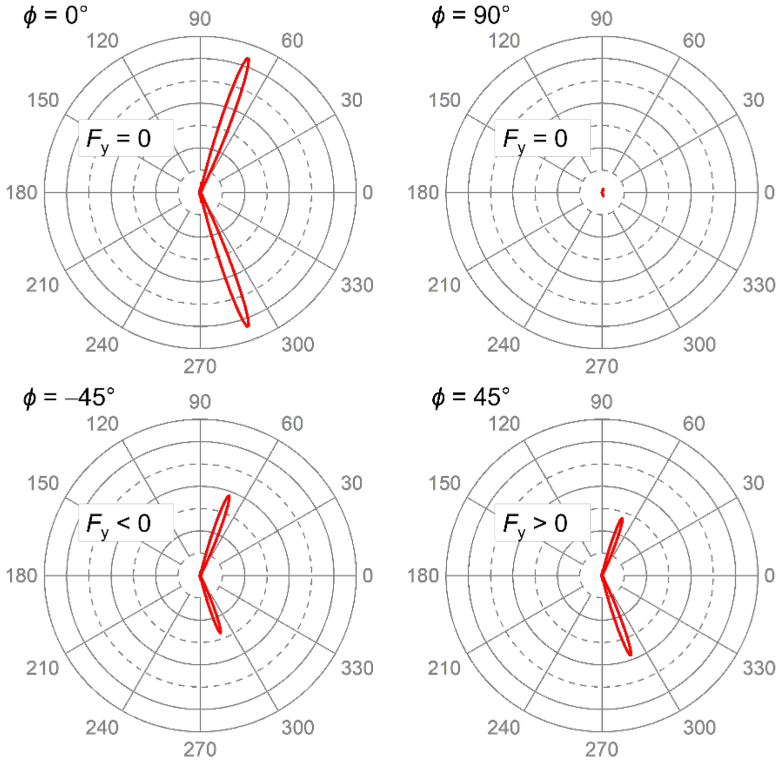

Supplementary Figure 5. Calculated far-field scattering patterns of a Ag nanocylinder for polarization angles ϕ = 0°, 90°, -45°, and 45°. The incident angle is 20°. The length and diameter of the Ag nanocylinder are 3900 and 80 nm, respectively. The Ag nanocylinder scatters more strongly with *p*-polarization (ϕ = 0°).

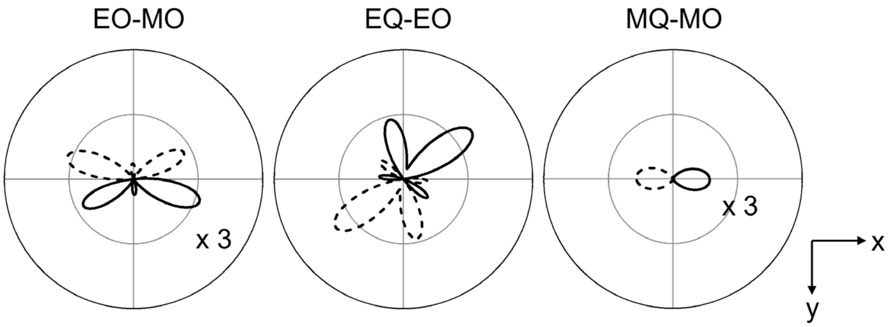

Supplementary Figure 6. Multipolar interplay analysis. A similar plot for the octupole-induced asymmetric radiation in the cylinder with L = 575 nm. EO, electric octupole; MO, magnetic octupole.

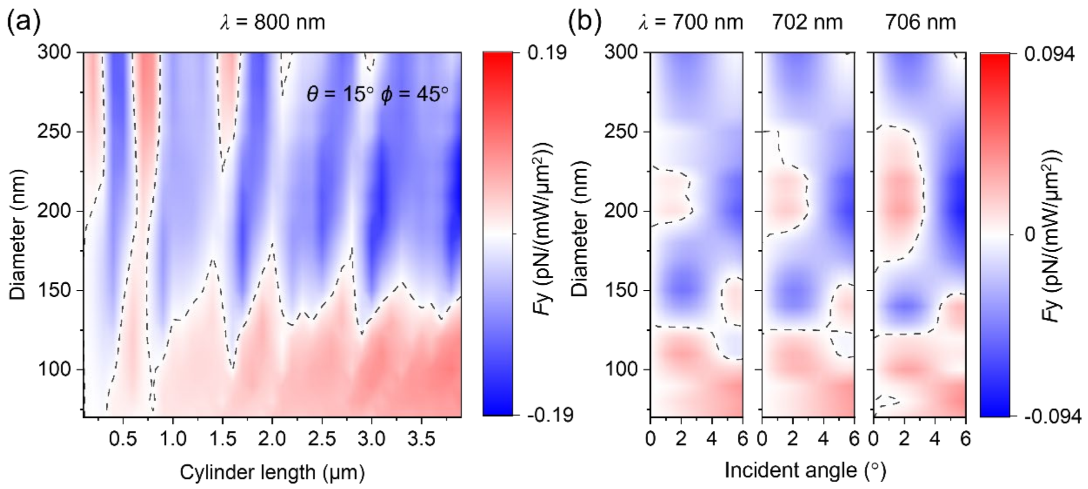

Supplementary Figure 7. Application for optical sorting. (a) Diameter- and length-dependent LOF. (b) Diameter-dependent LOF versus incident angle and wavelength (L = 3900 nm). The polarization angle is fixed at 45°.

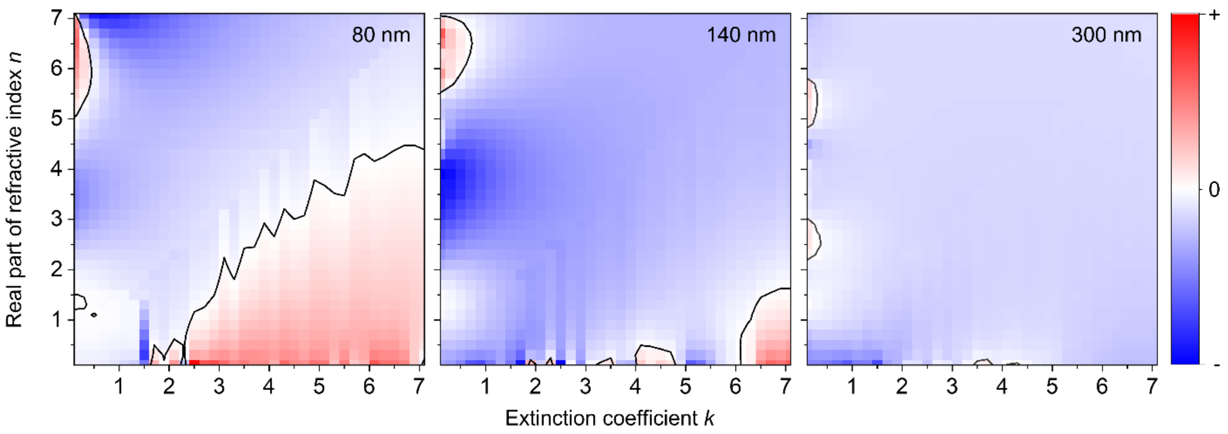

Supplementary Figure 8. Material-dependent LOF induced in a cylinder with different diameters. The length of the cylinders is 3900 nm. The incident angle is fixed at 2°.

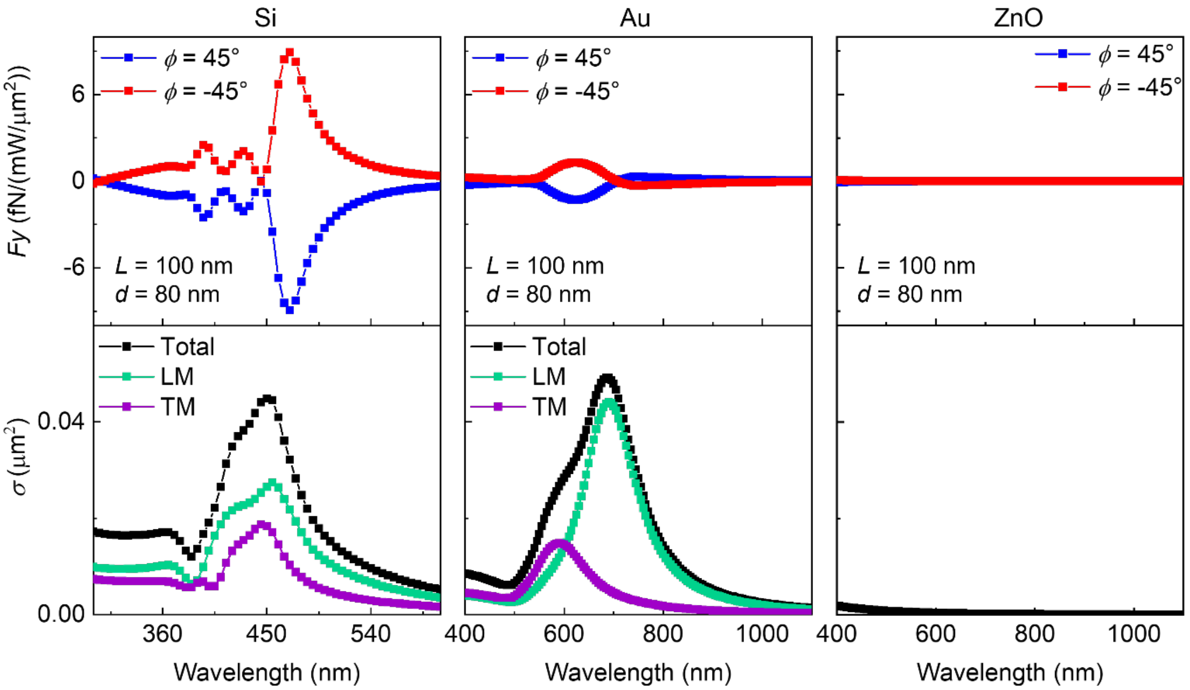

Supplementary Figure 9. Resonance-modulated LOF in a single Si nanocylinder. The diameter and length of the Si cylinder are 80 and 100 nm, respectively. LM and TM represent longitudinal and transverse mode, respectively. At the same time, the LOFs applied on Au and ZnO nanocylinders of the same size are presented. The role of the magnetic response in enhancing LOF is also clearly identified since Si particle can support a much stronger magnetic resonance than Au and ZnO.

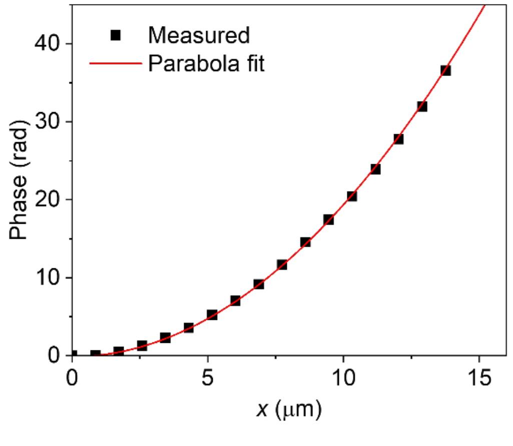

Supplementary Figure 10. The measured phase distribution of the FPOL. It can be fitted by a parabolic function $\varphi(x) = \xi x^2$, where x represents the spatial coordinates of the FPOL and ξ (0.2 rad/μm^2) controls the phase.

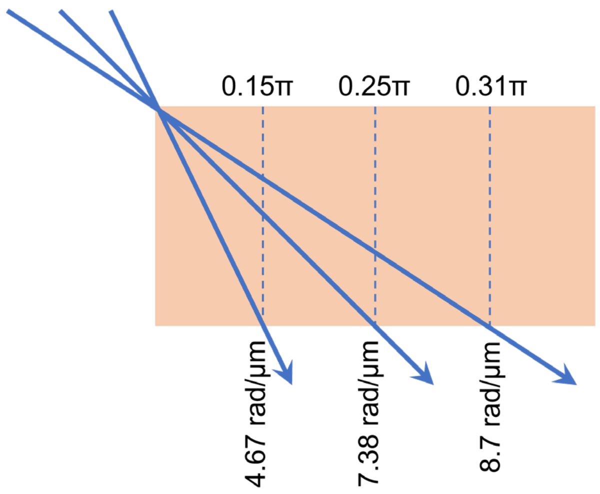

Supplementary Figure 11. Schematic of creating tunable optical phase gradient by a combination of inclined plane wave components. The incident angles of the three plane waves are 0.15π, 0.25 π, and 0.31π, respectively.

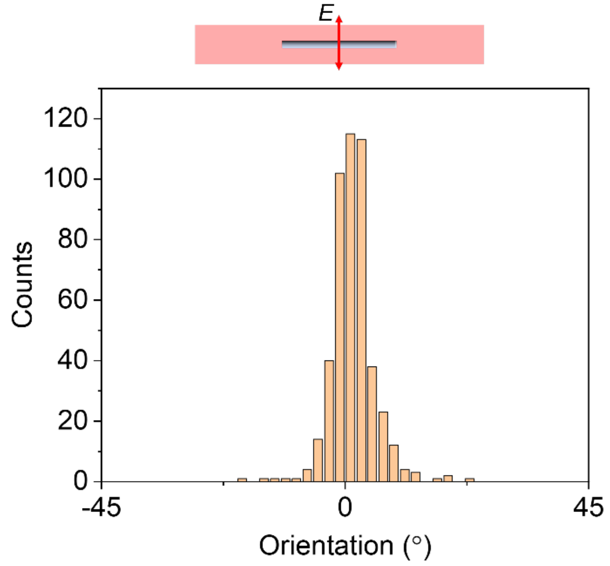

Supplementary Figure 12. Measured histogram of the orientation of the nanowire in the FPOL. The polarization direction is perpendicular to the orientation of the nanowire.

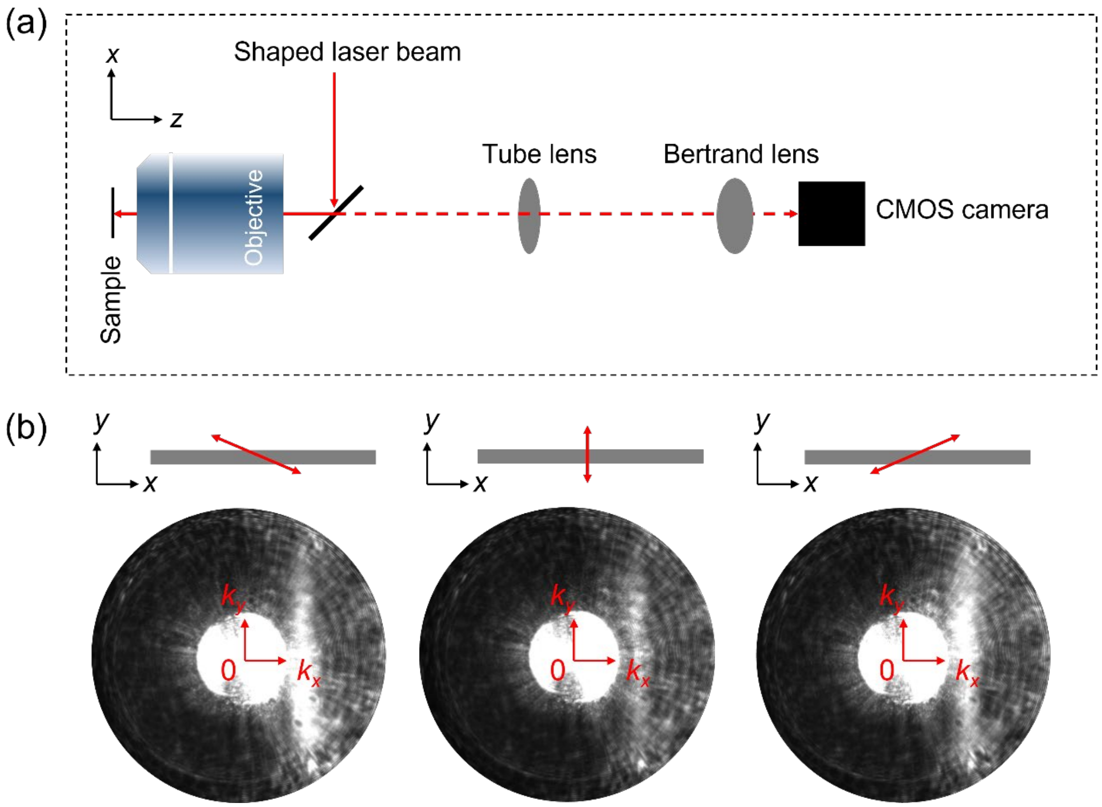

Supplementary Figure 13. Tuning of far-field scattering by polarization modulation. (a) The experimental setup. A Bertrand lens is removed or inserted before the microscope's image plane, which makes it possible to switch between real- and Fourier space imaging. (b) Measured full Fourier image of a single Ag nanowire under different polarization angles. Asymmetric scattering pattern is observed when the polarization angle is ± 30 degrees. The length of the nanowire is ~ 3.9 μm. The FPOL is orientated along *x*-axis. The numerical aperture of the objective is 1.2.

Supplementary Note 1. Optical rotation induced by linear polarization

To figure out if a nanorod could rotate, we checked the optical torque applied on a series of Ag nanocylinders with different lengths (their diameters are fixed at 80 nm). In the numerical calculations shown in the main text, the total optical force (F_y) is obtained by integrating the Maxwell stress tensor over a surface surrounding the whole cylinder. This surface can be divided into two equal parts, then the optical torque for the entire cylinder is the sum of the torque on each part relative to the cylinder center. When the polarization direction is parallel or perpendicular to the orientation of the cylinders, they do not experience any optical torque. When the polarization

angle is 45°, a cylinder with length of 100 nm shows a significant preference to align parallel to the polarization direction. As the cylinder length increases to 200 nm, it prefers to align perpendicular to the polarization direction. Fig. S14 shows the lateral force and torque applied on a long nanowire (L = 3900 nm) at different incident angles.

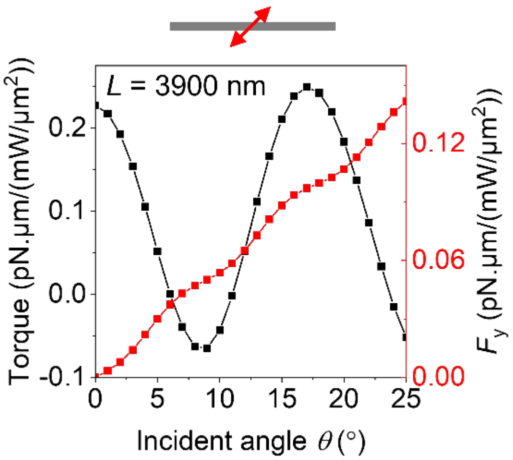

Supplementary Figure 14. Optical rotation induced by linear polarization. Calculated optical torque and lateral force of a long cylinder illuminated with different incident angles. The polarization angle is 45°. The cylinder' rotation direction may vary at different incident angles.

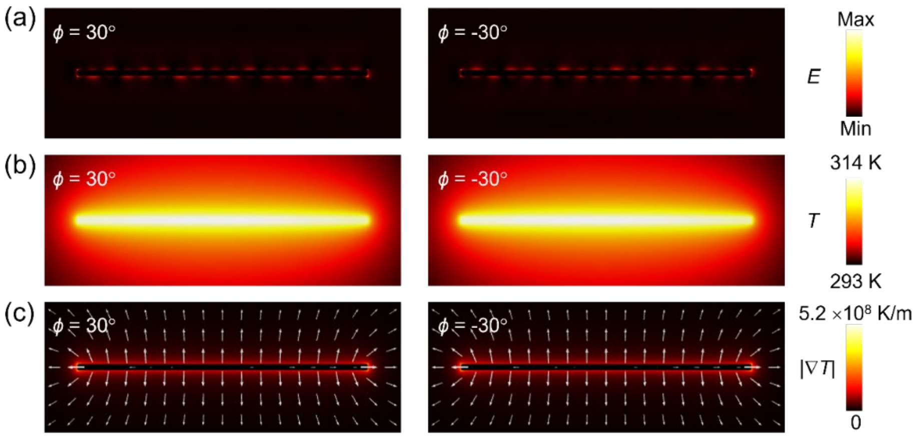

Supplementary Figure 15. Photothermal effect of a single Ag nanowire. (a) Calculated intensity distribution of the electric field with two different polarization angles. (b) Calculated temperature field distribution on the nanowire and the surrounding medium (x-y plane) corresponding to (a). (c) Simulated temperature gradient corresponding to (b). The white arrows represent the thermophoretic force vectors. The laser intensity is 4 mW/μm^2.

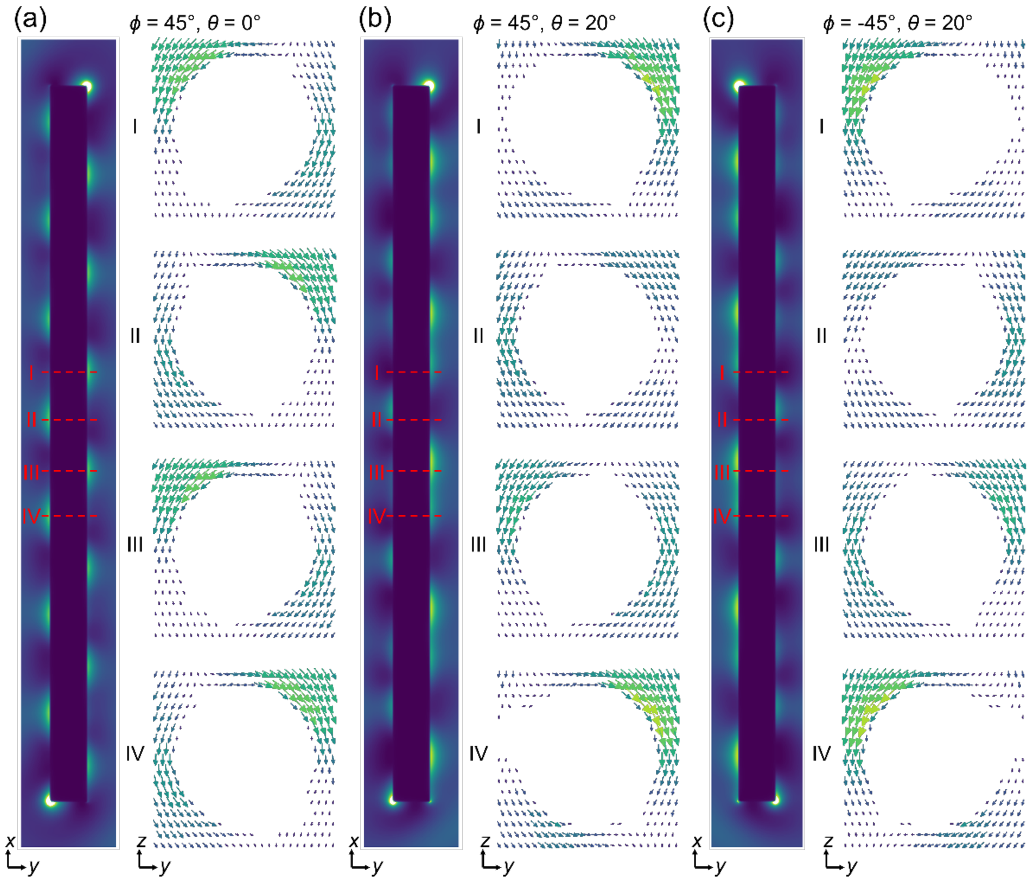

Supplementary Figure 16. Simulated intensity of the electric field and time-averaged Poynting vectors around single Ag microcylinders with (a) $\phi = 45°$ and $\theta = 0°$, (b) $\phi = 45°$ and $\theta = 20°$, and (c) $\phi = -45°$ and $\theta = 20°$. The left and right panels in (a-c) show the cross sections of the cylinder in the *x*-*y* (at $z = 0$) and selective *y*-*z* planes, respectively. The *x*-positions of these *y*-*z* planes are -0.35, -0.14, 0.14, and 0.35 μm, respectively. The diameter and length of the cylinder are 200 and 3900 nm, respectively.

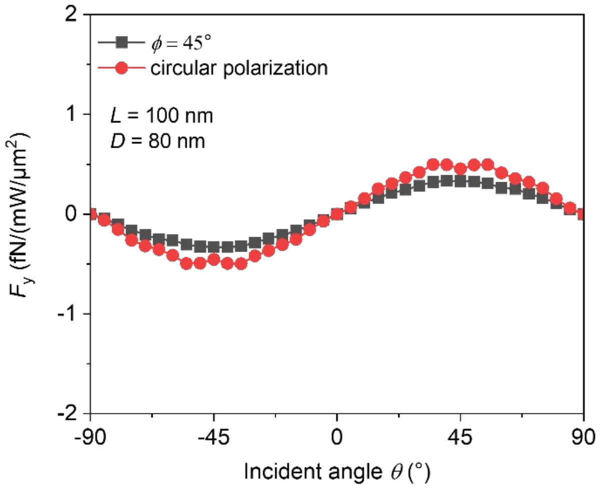

Supplementary Figure 17. LOF created by circular polarization. Calculated LOF in a short Ag nanocylinder with different incident angles and comparison to the corresponding LOF created by linear polarization.

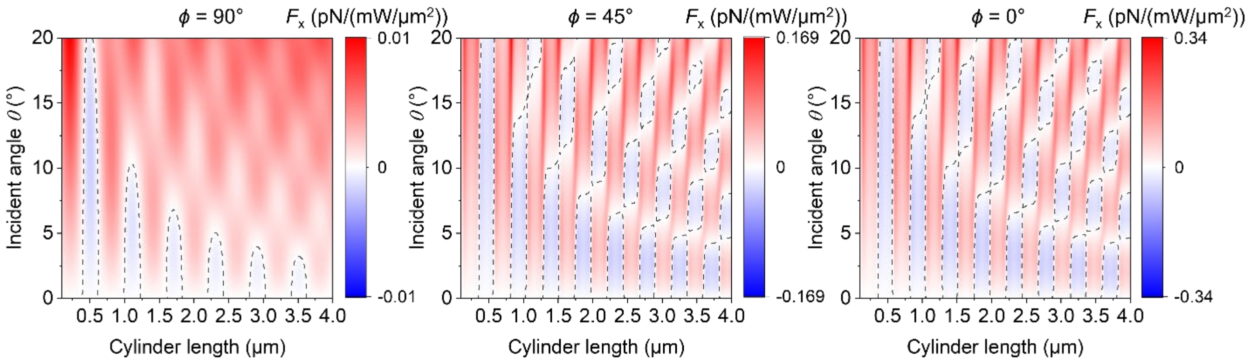

Supplementary Figure 18. Optical pulling and pushing of single Ag nanocylinders by a single plane wave. When the plane wave propagates in the *x-z* plane with incident angle θ (with respect to the *z* direction), the sign of optical force (F_x) can be positive (optical pushing) or negative (optical pulling) depending on the cylinder length and the incident and polarization angle. The contours denote $F_x = 0$. The diameter of the Ag cylinder is fixed at 80 nm. Strong optical pulling occurs at *p*-polarization ($\phi = 0°$). When the lateral optical forces are excited (e.g., ϕ changes from 0° to 45°), the optical pulling regions remain unchanged.

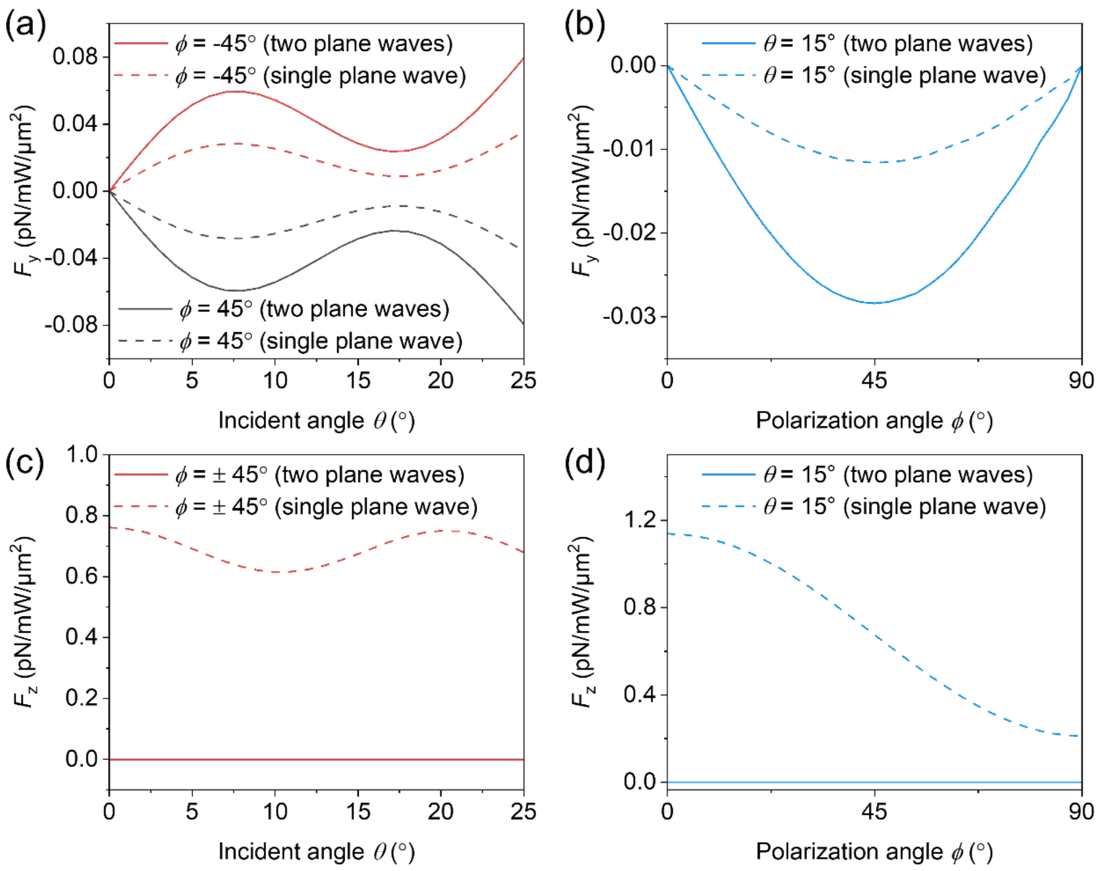

Supplementary Figure 19. Reversible and tunable LOF excited by two counter-propagated plane waves with linear polarization. (a) Calculated LOF (F_y) as a function of incident angle of two plane waves. The LOF takes opposite sign by switching the sign of the polarization angle ϕ. (b) Calculated LOF as a function of the polarization angle of an oblique plane wave (λ = 800 nm, θ = 15°). (c,d) The corresponding optical force (F_z) along z direction. The diameter and length of the Ag cylinder are 80 and 1500 nm, respectively. The F_y and F_z created by a single plane wave with the same incident and polarization angles are also shown for comparation (plotted by dashed lines).

(a)

Indices	$a_E(l, m) \times 10^7$	$a_M(l, m) \times 10^7$
$l = 1, m = -1$	$1.2273 + 0.9251i$	$0.0352 - 0.0636i$
$l = 1, m = 0$	$-0.0285 + 0.2221i$	$0.1388 + 0.0054i$
$l = 1, m = 1$	$-0.2324 - 0.7950i$	$-0.0474 - 0.0528i$
$l = 2, m = -2$	$0.6432 + 1.0280i$	$0.0444 + 0.0098i$
$l = 2, m = -1$	$-0.0261 + 0.0138i$	$0.0124 - 0.0927i$
$l = 2, m = 0$	$-0.5330 - 0.7461i$	$-0.0005 - 0.0055i$
$l = 2, m = 1$	$0.0277 + 0.0033i$	$-0.0110 + 0.0935i$
$l = 2, m = 2$	$0.6517 + 0.8005i$	$-0.0427 + 0.0052i$

(b)

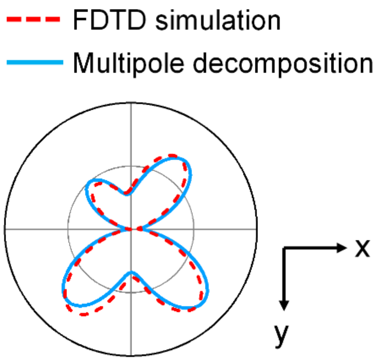

Supplementary Figure 20. Multipole expansion and reconstruction of the scattered field. (a) Calculated scattering coefficients for the cylinder with L = 350 nm and d = 80 nm. (b) Reconstructed radiation pattern in the x-y plane.
