# Supplementary material for: Creating tunable lateral optical forces through multipolar interplay in single nanowires
Source: Nat Commun. 2023 Oct 11;14:6361. doi: 10.1038/s41467-023-42076-x (PMC10567843; doi:10.1038/s41467-023-42076-x)
Supplement: Supplementary file 3 — Description of Additional Supplementary Files [file 41467_2023_42076_MOESM3_ESM.pdf]

Description of Additional Supplementary files

Supplementary Movie 1. Optical transportation and releasing of a single Ag nanowire with a polarization angle of -30 degrees. The nanowire moves along the optical line trap until it is released by the increment of a positive LOF.

Supplementary Movie 2. Optical transportation and releasing of a single Ag nanowire with a polarization angle of 30 degrees. The nanowire moves along the optical line trap until it is released by the increment of a negative LOF.

Supplementary Movie 3. Optical transportation of a single Ag nanowire with a polarization angle of 90 degrees.
